# Supplementary material for: Psychosocial problems, daily functioning and help-seeking behaviour of international migrant workers in the Netherlands: A qualitative study to inform the adaptation of a scalable stepped-care intervention
Source: Glob Ment Health (Camb). 2025 Dec 10;13:e2. doi: 10.1017/gmh.2025.10110 (PMC12766533; doi:10.1017/gmh.2025.10110)
Supplement: Roos et al. supplementary material [file S2054425125101106sup001.docx]

**Supplementary Table S1** *Daily Functioning Activities for Self-Care, Reported by International Migrant Workers in Free Listing Interviews (Round I), as Presented in the Focus Group Discussion (Round III)*

| **Activities to take care of themselves** | | |
| --- | --- | --- |
| Sports *(gym, running, soccer)* | Make new contacts | Meet up with friends |
| Enjoy the weather outside | Cooking | Watch movies |
| Take (online) courses | Go to spa/sauna | Learn to do your own manicure |
| Write a book | Go to hairdresser | Clean the house, do household chores |
| Art, drawing | Go get manicure/pedicure | Workshops |
| Winter swimming | Repair/renovate your home | Concerts |
| Improving IT skills; programming | Undergo medical examinations | Go to church |
| Watch tv | Get comfortable at home | Got to Saturday school |
| Go to cinema | Looking for extra income | Going on diet |
| Drink alcohol | Take extra vitamins | Visit the doctor |
| Exercise *(walk or cycle in the park)* | Follow the COVID rules (wash hands, wear face mask, 1.5m, see 1-2 person) | Get to know the country; visit cities/sea |
| Meditate | Socialize | Shopping |
| Eat healthy, drink a lot of water | Hobbies/leisure activities | Language courses |
| Applying for health insurance | Buddy system over Facebook | Open own business |
| Play PlayStation online | Read books | Living on your own; rent house |
| Walk the dog/play with pets | Dating/relationship | Clean yourself |
| Go to Polish shop and buy Polish food |  |  |

**Supplementary Table S2***Daily Functioning Activities for Family Care, Reported by International Migrant Workers in Free Listing Interviews (Round I), as Presented in the Focus Group Discussion (Round III)*

| **Activities to take care of their family** | |
| --- | --- |
| Send (expensive) gifts to family back home | Send letters |
| Send money to family back home (sometimes so much that they only keep enough money for rent and food) | Have online dinners (both eat the same food) |
| Video calls | Spend time with children |
| Work (so you can send money if your family needs it) | Spend time with elderly (walk with wheelchair) |
| Bring relatives to the Netherlands | Help out elderly people (do groceries) |
| Sending medication | Lie to family that everything is ok, even if it is not |
| Family comes and visits you in the Netherlands | Help each other (husband helps wife, wife helps husband) |
| Daily conversations | Phone talks |
| Offer unconditional support; you can always count on them | Visit your family back home |
| Home routines; help your wife at home (with the house chores) | Come to the Netherlands to get experience and new ideas so you can bring it back home and improve the family business |
| Come to the Netherlands to offer a better education for your children | Come to the Netherlands for good psychological wellbeing (because the Netherlands is in the top 10 happiest countries) |

**Supplementary Table S3***Daily Functioning Activities for Care of Family and Community, Reported by International Migrant Workers in Free Listing Interviews (Round I), as Presented in the Focus Group Discussion (Round III)*

| **Activities to take care of their friends/colleagues/community** | |
| --- | --- |
| Coffee take-away and go for a walk with friends | Meet up to cook and eat together |
| Open shops with food from their own country | Meet up to celebrate religions and talk about religion |
| Organize events | Make WhatsApp groups |
| Give classes, for example teach the local language to second generation citizens | Church (from your own country): if someone needs help, then the community helps him/her |
| Make online groups (e.g., Facebook) help each other; information exchange | Spend time with your close friends |
| Offer job if you have your own business | Help other people by buying groceries or moving houses |
| Bring traditional food from home country for colleagues | Listen to each other’s problems |
| Play sports | Online activities to meet new colleagues |
| Visit other places | Asking colleague to go for a walk |
| Play PlayStation together | Thematic WhatsApp groups (investing, movies, traveling etc.) |
| Drink together | Offer to drive someone to work |
| Meet up to play cards | Help someone with the job hunt |
| Look after each other’s children | Borrow money |
| Play board games | Invite Dutch people at your home |
| Colleagues: give birthday gifts | Translate for people who do not speak Dutch |
